# Single-Incision Laparoscopic Surgery for Intersigmoid Hernia

**DOI:** 10.1155/2014/589649

**Published:** 2014-11-19

**Authors:** Takahiro Watanabe, Hidetoshi Wada, Masanori Sato, Yuichirou Miyaki, Norihiko Shiiya

**Affiliations:** Division of General Surgery and Endoscopic Surgery, Surgery I, Hamamatsu University School of Medicine, 1-20-1 Handa-yama, Higashi-ku, Hamamatsu, Shizuoka 431-3192, Japan

## Abstract

Intersigmoid hernia is a rare form of internal hernia. Here, we report a case of intersigmoid hernia and provide a brief review of the 62 cases involving the mesosigmoid reported in Japan from 2000 to 2013. In the current case, a 26-year-old man with no previous history of abdominal surgery presented with abdominal pain and vomiting. Abdominal computed tomography revealed an extensively dilated small bowel and a closed loop of small bowel in the mesosigmoid. The patient was diagnosed with an intestinal obstruction due to an incarcerated internal hernia involving the mesosigmoid. There was no blood flow obstruction at the incarcerated bowel. An elective single-incision laparoscopic surgery was performed after decompression of the bowel using ileus tube. As the ileum herniated into the intersigmoid fossa, the patient was diagnosed with an intersigmoid hernia. The incarcerated small bowel was reduced in order to make it viable, and the hernial defect was closed with interrupted sutures. The patient had an uneventful recovery and was discharged on postoperative day five.

## 1. Introduction

Recently, laparoscopic surgery for acute abdomen, including intestinal obstruction, has become widely accepted among surgeons. Single-incision laparoscopic surgery is performed with an increasing frequency in many surgical centers. Although intersigmoid hernia is a rare form of internal hernia [1], this report presents a case of intersigmoid hernia and provides a review of the 62 cases involving the mesosigmoid reported in Japan from 2000 to 2013. In the current case, we preoperatively diagnosed an incarcerated internal hernia involving the mesosigmoid using abdominal computed tomography and went on to perform an elective single-incision laparoscopic surgery. The patient had an uneventful recovery.

## 2. Case Presentation

A 26-year-old man who presented with abdominal pain and vomiting was transferred to our hospital seven days after the onset of the symptoms. The patient had no history of abdominal surgery. On arrival, his vital signs were normal. Abdominal findings revealed distension and tenderness in the entire abdomen without any muscular defense signs. Laboratory examinations showed a white blood cell count of 7420/mm^3^, a C-reactive protein level of 0.3 mg/dL, and all other measured values within their normal limits.

**Figure 1:**
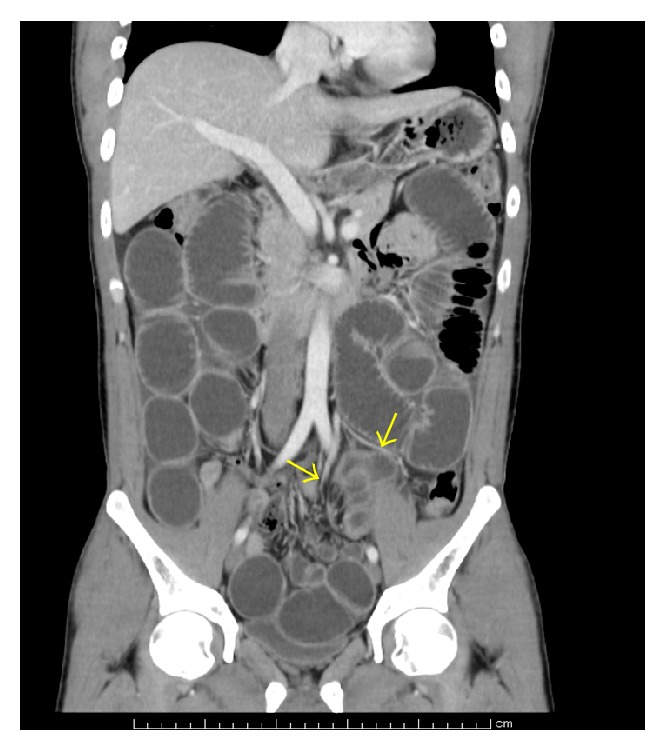
Computed tomography: abdominal computed tomography revealed an extensively dilated small bowel and a closed loop of small bowel in the mesosigmoid. The arrows indicate vessels of the mesosigmoid.

An abdominal X-ray examination showed a dilatation and an air-fluid level of the small bowel. Abdominal computed tomography revealed an extensively dilated small bowel and a closed loop of small bowel in the mesosigmoid (Figure 1). There was no blood flow obstruction at the closed loop of the small bowel. The patient was diagnosed with an intestinal obstruction due to an incarcerated internal hernia involving the mesosigmoid. An ileus tube was inserted to decompress the small bowel prior to an elective single-incision laparoscopic surgery that was performed six days after admission.

**Figure 2:**
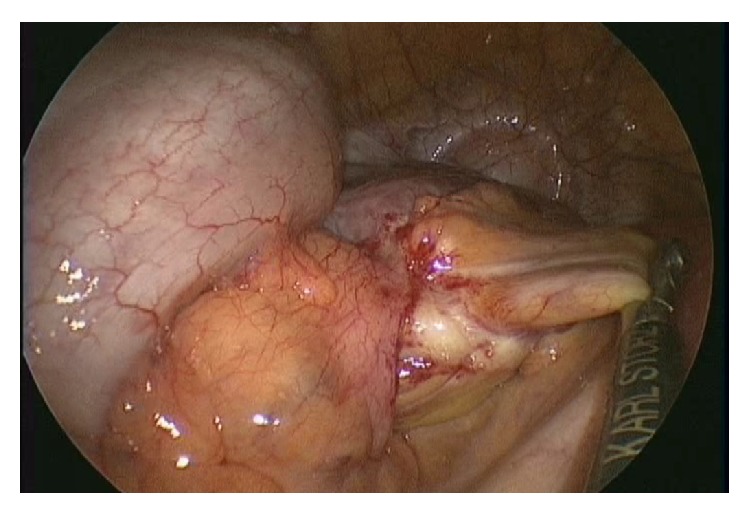
Intraoperative finding 1: the ileum herniated into the intersigmoid fossa. Mild adhesions of herniated ileum at the hernial orifice were observed.

**Figure 3:**
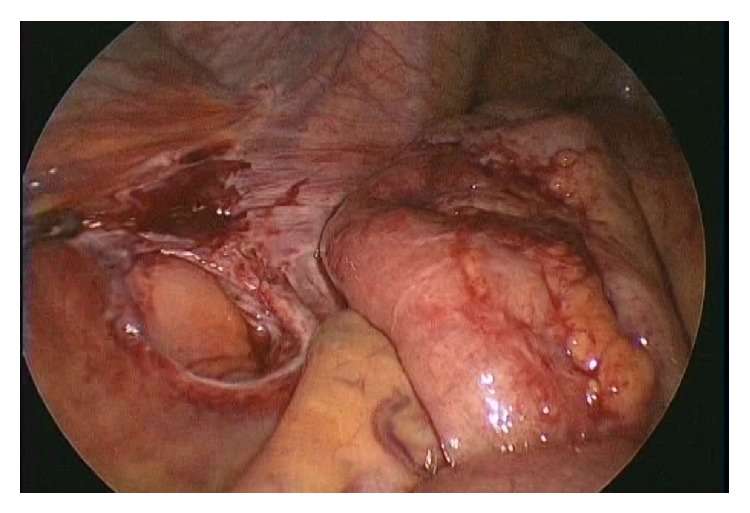
Intraoperative finding 2: the defect in the mesosigmoid was approximately 3 cm. The length of the incarcerated small bowel was approximately 10 cm. Redness can only be observed in the bowel.

**Figure 4:**
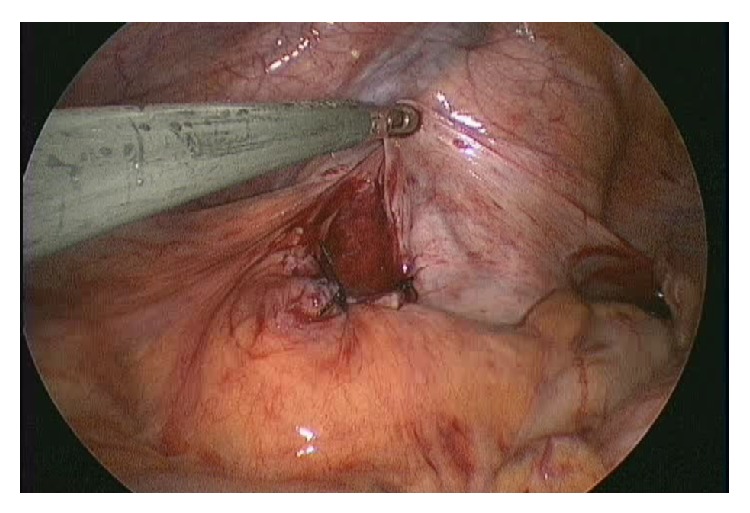
Intraoperative finding 3: the intersigmoid fossa was closed with interrupted sutures.

The operation was performed using a 2.0 cm transumbilical vertical incision. The fascia and peritoneum were then vertically opened, and three 5 mm trocars were inserted into the abdomen to introduce endoscopic instruments. Carbon dioxide was used to inflate the abdomen at 8 mmHg pressure. The operator used two 5 mm forceps, and the first assistant handled a 30 degree 5 mm laparoscope (Karl Storz, Tuttlingen, Germany). During the surgery, it was observed that the ileum had herniated into the intersigmoid fossa. Furthermore, there were mild adhesions of the herniated ileum at the hernial orifice (Figure 2). The incarcerated small bowel was reduced after adhesiolysis. Based on the intraoperative findings, the patient was diagnosed with an intersigmoid hernia. The defect in the mesosigmoid was approximately 3 cm, and the length of the incarcerated small bowel was approximately 10 cm. We confirmed that incarcerated small bowel was viable (Figure 3). The intersigmoid fossa was then closed with interrupted sutures (Figure 4). The patient had an uneventful recovery and was discharged on postoperative day five.

## 3. Discussion

Internal hernia is a rare cause of obstruction. Although the intersigmoid fossa is found in 65% of all autopsies [2], sigmoid mesocolon hernias account for only 6% of all internal hernias [1].

Benson and Killen [3] classified sigmoid mesocolon hernias as intersigmoid hernias, transmesosigmoid hernias, and intramesosigmoid hernias. While the current report involved a case of single-incision laparoscopic surgery for an intersigmoid hernia, a total of 62 cases of internal hernia involving the mesosigmoid have been reported in Japan from 2000 to 2013. Intersigmoid hernias accounted for 16 cases, transmesosigmoid hernias for nine, and intramesosigmoid hernias for the remaining 37 cases. A total of six cases involved bowel resection due to obvious intestinal necrosis. Out of those six cases, four were transmesosigmoid hernias, one was an intersigmoid hernia, and one was intramesosigmoid hernia. Fifteen cases were preoperatively diagnosed and accounted for 24% of all the cases. Out of these 15 cases, eight were intersigmoid hernias and seven were intramesosigmoid hernias. In the cases of intersigmoid or intramesosigmoid hernia, the possibility of intestinal necrosis is low. In conclusion, single-incision laparoscopic surgery is minimally invasive, provides excellent cosmesis, and is useful for intersigmoid hernia.
